# Evolution of Anemia Types During Antiretroviral Therapy—Implications for Treatment Outcomes and Quality of Life Among HIV-Infected Adults

**DOI:** 10.3390/nu11040755

**Published:** 2019-03-31

**Authors:** Amara E. Ezeamama, Alla Sikorskii, Ramanpreet K. Bajwa, Robert Tuke, Rachel B. Kyeyune, Jenifer I. Fenton, David Guwatudde, Wafaie W. Fawzi

**Affiliations:** 1 Department of Psychiatry, College of Osteopathic Medicine, Michigan State University, East Lansing, MI 48824, USA; Alla.Sikorskii@hc.msu.edu (A.S.); tukerobe@msu.edu (R.T.); 2 Department of Statistics and Probability, Michigan State University, East Lansing, MI 48824, USA; 3 College of Osteopathic Medicine, Michigan State University, East Lansing, MI 48824, USA; Bajwaram@msu.edu; 4 National Drug Authority, 23096 Kampala, Uganda; rbkyeyune@gmail.com; 5 Department of Food Science and Human Nutrition, Michigan State University, East Lansing, MI 48824, USA; imigjeni@msu.edu; 6 School of Public Health, Makerere University College of Health Sciences, P.O. Box 7062 Kampala, Uganda; dguwatudde@musph.ac.ug; 7 Departments of Global Health and Population, Nutrition and Epidemiology, Harvard T. H. Chan School of Public Health, Boston, MA 02115, USA; mina@hsph.harvard.edu

**Keywords:** HIV, anemia, anemia type, macrocytosis, microcytosis, anemia of chronic disease, ferritin, clinical outcomes, quality of life, antiretroviral therapy

## Abstract

This study examined whether the type of anemia in persons living with HIV/AIDS (PLWHA) changed from the beginning of highly antiretroviral therapy (HAART) and had implications for treatment outcomes and quality of life (QOL). If present, the anemia-type was defined as microcytic, macrocytic or anemia of chronic disease (ACD) at study months 0, 6, 12, and 18. Multinomial logistic regression quantified sociodemographic and HIV-treatment factors associated with incident microcytic anemia or ACD over 18 months. Repeated measures linear regression models estimated the anemia-type associated change in the CD4 cell-count, QOL, body mass index (BMI) and frailty over 18 months. Cox proportional hazard models estimated associations between anemia-type and time to (a) gain at least 100 CD4 cells/L and (b) hospitalization/death. Analyses were implemented in Statistical Analysis Software (v.9.4) from which odds ratios (ORs) mean differences (β) and corresponding 95% confidence intervals (CI) were estimated. At enrollment, ACD, macrocytic and microcytic anemia was present in 36.8% (*n* = 147), 11.3% (*n* = 45) and 9.5% (*n* = 38), respectively with 42% (*n* = 170) anemia-free. By the study end, only 23% (*n* = 115) were without anemia. Among the 251 with anemia at the study end, 53.3% (*n* = 195) had macrocytic anemia, 12.8% (*n* = 47) had ACD and 2.5% (*n* = 9) had microcytic anemia. Incident macrocytic anemia was positively associated with baseline hyperferritinemia (OR = 1.85, 95%CI: 1.03–3.32), inversely associated with wealth (OR = 0.87, 95%CI: 0.67–1.03) and inversely associated with efavirenz-containing HAART (OR = 0.42, 95%CI: 0.21–0.85). ACD incidence decreased by 53% (95%CI: 0.27–0.79) per 100 cells/L increase in baseline CD4-cell count and decreased by 90% (95%CI: 0.01,0.87) among adults treated with nevirapine-containing HAART. ACD was associated with a lower BMI at months 6 (β = −0.33, 95% CI: −0.64, −0.01) and 12 (β = −0.41, 95%CI: −0.73, −0.09), with lower QOL (β = −3.2, 95%CI: −5.94, −0.53) at month 12 and with elevated frailty (β = 1.2; 95%CI: 0.46, 1.86) at month 12. Macrocytic anemia did not predict a post-enrollment change in CD4, BMI or QOL during follow-up. However, the time to gain 100 CD4 cells/L was 43% slower (*p* < 0.05) and the frailty was higher at month 12 for PLWHA with the baseline or sustained macrocytic vs. no anemia. A substantial decline in ACD and microcytic anemia occurred in tandem with large increase in the macrocytic anemia over 18 months on HAART. Interventions to mitigate all anemia—particularly ACD, is expected to improve the immune recovery rate, lower frailty, and enhanced QOL.

## 1. Introduction

Anemia is by far the most common complication of HIV-infection [1,2,3]. Anemia has been associated with accelerated HIV disease progression and death among persons living with HIV/AIDS (PLWHA) [2] Treatment with highly active antiretroviral therapy (HAART) contributes to anemia resolution [4,5] due to the positive effect of HAART on the differentiation and survival of erythrocytes [4]. In spite of this, the overall burden of anemia in HAART treated PLWHA remains unacceptably high [4,6,7] and is associated with a low weight gain, slower immune recovery, low quality of life, hospitalization and death [3,4,7,8]. Whereas the persistent problem of anemia in individuals living with HIV is recognized, the etiology of anemia differs, may vary over time and effects on health outcomes may not be uniform by anemia type. 

An increase in the red blood cell mean corpuscular volume, i.e., the macrocytic cell picture, is a hallmark observation in patients treated with nucleoside reverse transcriptase inhibitors (NRTIs). Macrocytosis is also caused by vitamin B12 or folate deficiency [9,10,11]. The exact mechanisms leading to macrocytosis in HIV-infected patients treated with NRTIs is unknown [12,13]. NRTIs such as zidovudine are known to interfere with mitochondrial DNA synthesis through the inhibition of polymerases needed for erythrocyte formation. Interference with mitochondrial DNA synthesis impairs the erythrocyte cell division and ultimately threatens cell survival and viability [12,13]. For the most part, macrocytosis in treated HIV-infections is thought to be benign; however, at least one study has challenged this notion by linking NRTI-induced macrocytosis to mitochondrial toxicity [14]. 

One study of zidovudine-treated HIV-infected patients with macrocytosis demonstrated that macrocytosis was sustained for at least two years following the cessation of zidovudine treatment in 37% of the patients but associations between sustained macrocytosis and morbidity were not specifically explored [15]. Among PLWHA, low vitamin B12 or folic acid levels were not observed in patients with macrocytosis [16]. However, clinical investigations have often linked HAART regimen with resolution or exacerbation of different forms of anemia [4,17,18]. For example, a forty-fold increase in the risk of macrocytosis was noted for PLWHA treated with zidovudine compared to patients who did not use zidovudine [13]. Similarly, treatment with efavirenz was associated with hemolytic anemia among HIV patients due to cell shrinkage [19,20]. Therefore, it is thought that certain types of anemia, especially macrocytic anemia, is more likely associated with treatment rather than the HIV disease process [12]. There is limited specific data on the types of anemia present in PLWHA in the beginning on HAART, how the anemia etiology in this vulnerable group may vary during HAART, and the implication of changing anemia types for immune recovery, clinical outcomes (weight gain, frailty-related phenotype, hospitalization/death) and patient-reported outcomes such as quality of life. 

To address the existing knowledge gaps, we report on changes in the anemia type and associations with morbidity in a sample of adult PLWHA just initiating HAART or who have initiated HAART within the past 6 months. These PLWHA participated in a recently completed trial of multivitamins B, C, E vs. the placebo and were followed up at 6, 12, and 18 months. We have previously reported that 49% of this sample had WHO age-sex defined anemia at the HAART initiation [7]. Of those with anemia at enrolment, more than 63% remained with anemia over 18 months of the follow-up [7]. This study tests the hypothesis that severity of adverse immune, clinical and patient-reported outcomes experienced over time on HAART will vary by type of anemia by exploring the following aims:

1. To determine the prevalence of anemia of various types at the baseline, and at 6, 12 and 18 months of the follow-up.
2. To identify associations between baseline behavioral, sociodemographic and clinical parameters, and the development of specific types of anemia among PLWHA without anemia at enrolment.
3. To identify associations between anemia types, and changes in body mass index, CD4-cell count, QOL and Frailty at 6, 12, and 18 months follow-up.
4. To identify associations between anemia types, and time to the post-enrolment gain of ≥100 CD4 cells, hospitalization or death.

## 2. Materials and Methods 

### 2.1. Study Population and Design

Data for this study arose from a multivitamin supplementation trial (single recommended daily allowance (RDA) of vitamins B, C and E vs. placebo) conducted among HIV-infected Ugandan adults between 2009 and 2012. All participants had a World Health Organization (WHO) HIV disease stage 3 or higher, were initiating HAART upon study enrollment or had been on HAART for six months or less. Participants were followed-up every 6 months for a total of 18 months. There were no intervention-related differences in CD4 cell counts, weight or quality of life (QOL) improvements noted over the 18 months of follow-up [21]. All analyses were adjusted for the allocated intervention during the trial.

Consent Process/Ethical Approval: At enrollment in the parent study, each participant provided written informed consent administered in the local language of Luganda. The study was approved by the Scientific Review Committee of the Infectious Diseases Institute at Makerere University College of Health Sciences; and the Institutional Review Boards of Harvard School of Public Health (protocol number: 17361) and that of Makerere University School of Public Health (protocol number: HDREC 067). The study was further registered and approved by the Uganda National Council for Science and Technology (UNCST, protocol number: HS 629). 

### 2.2. Measurements

Continuous Outcome Measures: CD4-cell count, Body Mass Index (BMI), QOL and Frailty

Venous whole blood in lavender top tubes was extracted at each study interval (months 0, 6, 12, 18) for laboratory assessment of complete blood count (CBC) and absolute T-cell lymphocyte count (CD4) for each participant. Absolute T-cell lymphocyte count in cells/microliter was measured using a FACS Calibur flow cytometer (Becton-Dickinson, San Jose, CA, USA). Body mass index (BMI) was calculated as the ratio of objectively measured weight (in kilograms) to squared height (in meters). QOL was assessed with the Medical Outcomes Study HIV Health Survey translated and culturally adapted for the study area [22]. As described previously [23], an overall QOL score was calculated as the sum of scores for activities of daily living, cognitive function, emotional and physical health subscales. Frailty score is defined as in earlier work by our team [6] and reflects a state of decreased physiologic reserves corresponding with increasing disability and higher morbidity and mortality risks [24,25] including unintentional weight loss, decreased level of physical activity, slowness, weakness and exhaustion. Overall QOL and frailty score were linearly transformed so that the lowest possible score would be zero and the highest possible score would be 100.

*Dichotomous Outcomes/Events:* CD4 cell count gain greater than 100 cells/L, Death/Hospitalization

CD4 cell count gain greater than 100 cells/L: At each follow-up interval (months 6, 12 and 18), respondents were evaluated for occurrence of CD4 gains greater than 100 cells/L from baseline. Observation times were censored at the follow-up date on which this event occurred. For participants that did not experience CD4 cell counts beyond this magnitude, censoring was at the study end or at the last observation period, if lost to follow-up. 

Death/hospitalization: Occurrence of hospitalization or death was for each participant. Censoring was at the date of first hospitalization or death. Participants that experienced both events were censored at hospitalization. 

Anemia Type/Etiology: Anemia type was assessed four times (months 0, 6, 12 and 18) and defined using a combination of WHO age/sex-specific hemoglobin thresholds (i.e., hemoglobin <13 g/dL for men and <12 g/dL if female) and abnormal components of CBC including mean corpuscular volume (MCV), mean corpuscular hemoglobin (MCH) and mean corpuscular hemoglobin concentration (MCHC). Four types of anemia were defined with dysregulated levels of MCV, MCH and MCV defined as in previous work among HIV-infected Ugandan adults [26] as follows: 

Microcytic Anemia: included persons with classic iron deficiency anemia defined operationally defined as low MCV (<73 femtoliters (fl)) or low MCH (i.e., <24 picograms (pg)). 

Macrocytic Anemia: identified persons with anemia potentially due to folate or vitamin B-12 deficiency and defined by high MCH (i.e., >35 pg) or high MCV (i.e., >99 fl).

Anemia of Chronic Disease (ACD): included non-macrocytic and non-microcytic anemia—also known as anemia of inflammation or normocytic anemia where age/sex-specific hemoglobin is low in the presence of normal CBC indices (i.e., normal MCV, MCH and MCHC). 

No Anemia of any type: individuals without microcytic, macrocytic or ACD were classified as anemia free. 

Change in Anemia Type: Time-varying anemia type was assessed in two ways in relationship to post-baseline morbidity outcomes [1]. Current Anemia type (in the four categories described above) was related to change in CD4, QOL, frailty and BMI. For each individual, the anemia type value varied over time and the anemia type at each follow-up interval were associated with the respective outcomes [2]. Six summarized patterns of change in the anemia type (a-f below) up to the event occurrence or at the censoring interval were defined separately for analyses of (a) gain of >= 100 CD4 cells and (b) hospitalization or death.

(a) Baseline ACD/microcytosis sustained through one or more follow-up.
(b) Baseline ACD/microcytosis resolved in follow-up but replaced with macrocytosis.
(c) Macrocytosis at baseline sustained in all follow-up intervals.
(d) New ACD/microcytic anemia among participants without baseline anemia.
(e) New macrocytosis among participants without baseline anemia.
(f) No anemia at baseline and in follow-up intervals.

For each outcome, patterns (a–d) were compared to anemia free individuals (f) in all analyses. 

### 2.3. Potential Confounders: Clinical, Socio-Demographic and Behavioral Characteristics 

Age was measured in completed years and categorized by the approximate quartiles of age distribution in our sample as 18–29, 30–35, 36–41 and 42+. Socioeconomic status was defined by years of education, employment status and household wealth or assets owned. Composite household wealth scores were categorized at quintiles [27]. History and current use of alcohol (former, current or never drinker) and ever vs. never cigarette smoking were self-reported. 

### 2.4. Statistical Analysis

To address Aim 1, the number and proportion of respondents within each category of anemia type at enrollment were calculated and sociodemographic behavioral and clinical characteristics of the enrolled participants were summarized by anemia type at enrollment. Chi-square tests were used to test the differences in these characteristics across anemia types at the baseline. Next, the number (and proportions) of persons affected by various types of anemia were evaluated at 6, 12, and 18 months. 

To address Aim 2, the first set of multivariable analyses was designed to determine baseline factors- including trial arms, associated with developing one of three types anemia over 18 months of follow-up in PLWHA without anemia at enrolment. In these analyses, anemia was a multinomial outcome with four levels (microcytic, macrocytic, ACD or no-anemia), hence, we implemented longitudinal multinomial logistic regression analyses using generalized estimating equations (GEE) modeling in SAS 9.4 GEE procedure. No anemia was the constant reference category at all time points. The GEE model quantified the odds of incident ACD, or Macrocytic anemia in the relationship to the baseline behavioral, sociodemographic and clinical parameters. Because of the post-baseline incidence of microcytic anemia (<3%, *n* = 12 persons maximum), there was limited statistical power to quantify the determinants of microcytic anemia in this sample. This anemia type was not included in GEE modeling.

To address Aim 3, the morbidity impact of anemia type defined at the baseline and within each follow-up interval (i.e., current anemia type at months 6, 12 and 18) were investigated as determinants of each continuous outcome (CD4-cell count, BMI, QOL and Frailty). Outcomes were assessed at months 0, 6, 12 and 18 and separate multivariable that repeated the measures of linear regression analyses were implemented using the SAS Mixed Procedure. These analyses were adjusted for baseline socio-demographic characteristics (age, sex, wealth), nutritional status (trial arms, baseline prevalent multivitamin use, vitamin D status), general immune activation (CRP) and baseline HAART status as potential confounders established per subject matter knowledge in light of the literature and bivariate analyses where *p*-values <=0.20. For each outcome measured, the anemia type-related differences in the adjusted means and corresponding 95% confidence intervals were computed. 

To address Aim 4, the multivariable Cox proportional hazards regression using PROC PHREG in SAS was implemented to quantify the baseline anemia type and change in anemia-type-related differences in the time for (a) a post-enrollment gain of more than 100 cells/L and (b) the time to hospitalization/death as a composite outcome. These models were adjusted for age, sex, wealth, baseline CRP, baseline HAART status, multivitamin use history, baseline vitamin D, and smoking status. 

## 3. Results

### 3.1. Baseline Characteristics 

This secondary analysis included 400 HIV-positive adult Ugandans, including 275 women and 125 men, previously enrolled in a micronutrient trial of multivitamins BCE vs. placebo. At enrollment, 42% (*n* = 170) of study participants were anemia free. The prevalence of anemia of chronic disease (ACD), macrocytic and microcytic anemia were 36.8% (*n* = 147), 11.3% (*n* = 45) and 9.5% (*n* = 38), respectively. There were no systematic differences across the baseline anemia type with respect to age, smoking, alcohol use, self-reported multivitamin use, and educational level. Microcytic anemia at enrolment occurred at a higher frequency among PLWH with any (12.5%) vs. no (6.5%) HAART experience at enrolment (Table 1 and Table 2). Similarly, the proportion of HAART naïve individuals, individuals with hyperferritinemia and male PLWH was higher among individuals with baseline ACD. Macrocytic anemia at enrollment was positively associated with participant reported multivitamin use and a higher prevalence of vitamin D deficiency/insufficiency (Table 1). 

### 3.2. Change in Anemia Type Over the 18-Month Follow-up

Over the 18-month study period, anemia remained highly prevalent in the sample but the type of anemia evolved significantly. Specifically, the prevalence of microcytic anemia declined steadily from 9.5% (*n* = 38) at enrollment to 2.5% (*n* = 9) by month 18. Likewise, the prevalence of ACD declined steadily from a baseline prevalence of 36.8% at enrolment to 12.8% by study month 18. Macrocytic anemia, on the other hand, increased from a baseline prevalence of 11.3% to a 53.3% prevalence by study end. The trend in anemia type prevalence over the 18-month follow-up was similar to the baseline HAART status (Table 2). Among participants free of anemia at the baseline (*n* = 170), four new episodes of microcytic anemia developed in two participants, 20 total new episodes of ACD developed in 14 individuals and a total of 284 macrocytic anemia episodes developed in 109 individuals between study months 6 and 18 (data not shown). 

**Baseline Predictors of Incident Macrocytic Anemia:** Participants treated with efavirenz relative to those HAART naïve at enrolment were less likely (OR = 0.42, 95% CI: 0.21, 0.85). Similarly, the odds of developing macrocytic anemia declined about 17% per quintile increment in household wealth but this association was significant for only one of four lower quintiles vs. the highest wealth quintile. On the other hand, the odds of incident macrocytic anemia was elevated (OR = 1.85, 95%CI:1.03, 3.32) for individuals with high vs. normal baseline ferritin. Compared to individuals randomized to placebo, those randomized to BCE had marginally higher odds (OR = 1.86, 95%CI: 0.99, 3.51) of developing macrocytic anemia over the study period. There were no age, sex, baseline CD4 and baseline weight-related differences in the onset of macrocytic anemia over 18 months. (Table 3)

**Baseline Predictors of Incident ACD:** Among participants free of anemia at enrolment, relative immune competence at the study initiation and the type of HAART regimen compared to HAART naïve status were associated with the development of ACD over the study period. Specifically, per 100 cells/L increase in CD4 cell count, the odds of developing new ACD over the follow-up declined by as much as 53% (95% CI: 0.27, 0.79). Similarly, nevirapine therapy relative to HAART naïve status was associated with a 90% lower likelihood of developing ACD (Table 3). 

There was no association between ACD onset over the 18-month follow-up and the following factors: age, high/low vs. normal ferritin status, micronutrient supplementation with BCE vs. placebo, baseline BMI and wealth. Although the associations were not statistically significant, the following were associated with higher odds of developing ACD over the 18-months of follow-up: female vs. male sex (OR = 3.6, 95% CI: 0.71, 18.3), underweight vs. normal weight (OR = 2.19, 95% CI: 0.52, 9.12) and vitamin D deficient vs. sufficient status (OR = 6.7, 95% CI: 0.52, 86.3). 

### 3.3. Associations between Anemia Type and Change in CD4, BMI, QOL and Frailty: 

Among enrolled participants, change in absolute CD4 count (*p*-value: Anemia type × Time ≤ 0.0077) and BMI (*p*-value: Anemia type × Time ≤ 0.0852) over 18 months varied by anemia type whether define at the baseline or current anemia type. At the baseline, CD4 cell counts were similar for adult PLWHA with microcytic or ACD compared to those without anemia. At all follow-up intervals, there was no evidence of an anemia-type related difference in mean CD4 cell count. On the other hand, adult PLWHA with macrocytic vs. no anemia at enrollment started had a higher baseline CD4 cell count (difference: 43.0, 95% CI: 13.4, 72.1). However, this initial macrocytic anemia related CD4 advantage in comparison with no anemia is absent in all follow-up intervals whether macrocytic anemia is defined at baseline or per value at current follow-up interval. 

There is no evidence of temporal variations over 18 months for the baseline or current anemia-type related change in QOL (*p*-value: anemia type × time ≤ 0.4860) and frailty-related phenotype (*p*-value: baseline anemia type × time ≤ 0.3616). At baseline, the average QOL score was lower for participants with ACD (Difference: −3.0, 95%: −4.9, −1.1) and for those with microcytic anemia (Difference: −3.7, 95%CI: −6.8, −0.6) compared to participants without anemia. At each follow-up interval, microcytic anemia and ACD were each associated with lower QOL although statistical significance was evident at Month 12 for comparison of ACD vs. no anemia only. Further, ACD was associated with the elevated frailty in three of four follow-up periods: at months 0 (*p* < 0.05), at month 6 (*p* = 0.057) and at month 12 (*p* < 0.05). Macrocytic vs. no anemia status was not associated with BMI or QOL. However, macrocytic vs. no anemia was associated with elevated frailty at the follow-up month 12. At month 18, there was no anemia-type related differences in CD4, BMI, QOL and frailty-related phenotype (Table 4).

Time to the post-enrolment gain of 100 CD4 cells/L was 43% slower for individuals that began the study with macrocytic anemia vs. no anemia. Baseline microcytic and ACD vs. no anemia were not associated with the speed of immune recovery. Analyses of patterns of change in the anemia type in the relationship to the immune recovery rate finds significantly slower immune recovery for participants with sustained ACD/microcytosis (HR = 0.57, 95%CI: 0.36, 0.89) vs. no anemia and for those with sustained macrocytosis (HR = 0.43, 95%CI: 0.25, 0.73) from baseline vs. no anemia. Among persons who were anemia free at the baseline, incident macrocytosis (HR = 0.77, 95% CI: 0.52, 1.14) vs. no anemia and incident ACD (HR = 0.52, 95% CI: 0.25, 1.08) vs. no anemia were each associated with slower immune recovery though these associations were not statistically significant. There were no baseline anemia type or pattern of change in anemia type related differences in hospitalization or death over the study period (Table 5).

## 4. Discussion

In line with our hypothesis, the type of anemia present at or near the beginning of HAART evolved significantly over 18 months of antiretroviral therapy among adult PLWHA from Uganda. At an 18-month follow-up, an 82% decline in ACD and a 71% decline in microcytic anemia was accompanied by a striking 370% elaboration in macrocytic anemia type. This finding is consistent with the previously reported 17.5% to 37% decline in ACD and 53% to 84% decline in microcytic anemia matched with a 513% to 940% elaboration of macrocytic anemia in African adult PLWHA followed for either 6 months [4] or 96 weeks [28] post-HAART initiation. Unlike these prior investigations [4,28], the baseline pre-HAART prevalence of macrocytic anemia in our study was four times larger than previously noted among adult PLWHA from Uganda and Ethiopia. Among PLWHA, certain HAART regimens, particularly nucleoside reverse transcriptase inhibitors such as AZT/zidovudine, stavudine (d4T) and 3TC [12] have been associated with macrocytosis. Of note, this sample included 50% PLWHA just starting at HAART and another 50% that had been on HAART for a median duration of 0.2 months prior to recruitment for the parent study—a multivitamin trial [29,30,31,32]. If HAART induced macrocytosis occurs relatively close in time to the beginning of HAART, the elevated baseline prevalence of macrocytosis in this cohort could be a reflection of the short period of HAART experienced in half the sample. The exact mechanisms by which certain HAART regimen induce macrocytosis are incompletely elucidated but interference with DNA red blood cell synthesis and maturation has been reported [12,13,33]. In addition, commonly co-administered drugs in PLWHA such as Co-trimoxazole/dapsone given as prophylaxis against opportunistic infections antagonize dihydrofolate reductase and dihydropteroate synthase (a rate-limiting step enzyme in folate metabolism) leading to macrocytic anemia [13]. Furthermore, macrocytic anemia may also arise from folate/vitamin B-12 deficiency contributing to the noted profound shift from predominantly baseline ACD or a no anemia baseline to macrocytic anemia during the follow-up period as previously reported in one study from a low-income country [34]. 

Among adult PLWHA anemia free at enrolment in this sample, the development of macrocytic anemia was inversely associated with the use of efavirenz-containing HAART suggesting variations in the HAART regimen with respect to the elaboration of macrocytosis. In addition, household wealth is protected against the onset of macrocytosis. This relationship, if true, may suggest that socioeconomic status-related differences in nutritional quality—specifically, diets low in vitamin B-12 and folate—contribute to the elaboration of macrocytic anemia. Of note, this study uses data arising from a multiple micronutrient supplementation trials of one recommended daily allowance of vitamins B, C, and E vs. the placebo. Supplementation would be expected to protect from macrocytosis for PLWHA allocated for this intervention, but this was not observed. Other studies among adult PLWHA has found no association between macrocytosis and folate or vitamin B-12 deficiency [17,35,36]. We did not specifically measure folate and vitamin B-12 levels in this study and are thus unable to further clarify the contribution of nutritional deficiency to the substantial increase in macrocytic anemia observed over 18 months of HAART. 

While macrocytic anemia due to HAART has been described, its morbidity relevance among PLWHA is unknown. At least four observations in this sample suggest that the elaboration and/or persistence of macrocytic anemia in adult PLWHA may not be innocuous with respect to morbidity. First, the proportion of adults with HAART experience was similar across anemia-types at enrolment, suggesting that macrocytosis-related higher CD4 at enrolment may not be entirely explained by variations in HAART experience. Second, macrocytosis in this sample was associated with higher frailty in all three follow-up intervals with statistical significance achieved in one interval. Third, among adults without baseline anemia, the development of macrocytosis was associated with slower, though not statistically robust, immune recovery. Fourth, the noted significant association between sustained macrocytosis and slower immune recovery suggests this form of anemia may contribute to disability and/or other physiologic vulnerabilities among PLWHA on HAART. 

The pre-HAART microcytic anemia prevalence in this study is similar to the 14.9% observed in Ethiopian adults but less than the 30% previously reported in Ugandan adult PLWHA [28]. Among participants free of anemia at the baseline, the development of microcytic anemia post-HAART initiation was rare and inversely associated with CD4 cell count and treatment with nevirapine-based HAART. Among adult PLWHA that began HAART with any type of anemia on the other hand, the finding that ACD was the dominant type of anemia (present in 64% of anemics) before beginning HAART is also consistent with reports from Ethiopia [37] and Uganda [28]. The decreasing prevalence of ACD and microcytic anemia during follow-up is consistent with the demonstrated positive impact of HAART on resolving these forms of anemia through HIV viral suppression, reduction of HIV related inflammation or interruption of dysfunctional erythropoiesis at the level of the bone marrow [38]. As expected, starting HAART with ACD was associated with less weight gain, had lower QOL and elevated frailty during follow-up. These findings highlight the morbidity consequences of ACD which is highly prevalent pre-HAART. Morbidity due to ACD remains a continuing concern in PLWHA as 13% to 19% of PLWHA had ACD during follow-up, likely as a function of low-level chronic inflammation despite HAART. 

The main limitations of this study that should lead to the cautious interpretation of results are the lack of serum measures of folate/vitamin B-12 to clarify the contribution of nutritional anemia to observed morbidities. Future longitudinal studies that include measures of folate/vitamin B12 designed to investigate the morbidity relevance of macrocytosis in PLWHA will be important to confirm or refute these observations. Strengths of the present study that enhance causal inference for the relationship of the anemia type to respective outcomes include longitudinal study design, robust analytic strategy, robust control for key confounders and repeat assessments of anemia type and respective outcome measures. 

## 5. Conclusions

In summary, over 18 months on HAART in this sample of adult PLWHA from Uganda, the vast majority of PLWHA remained anemic with dynamic changes in the type of anemia over time. We confirm HAART related reductions in microcytic and ACD type anemia over time as well as the striking elaboration of macrocytic anemia. While ACD resolves over time, it remained highly prevalent among adult PLWHA despite HAART and developed for the first time in 8% of PLWHA anemia free at enrolment. Hence, efforts to minimize post-HAART ACD are warranted with possible benefits for an improved immune recovery rate, lower frailty and enhanced QOL. For the first time, the elaboration of microcytosis is associated with slower immune recovery and elevated frailty. Future longitudinal investigations of potential macrocytosis related differences in long-term neurocognitive and clinical outcomes are needed to further clarify the role of this form of anemia in the context of multi-decade HAART for millions of PLWHA. Given the dramatic elaboration of HAART over time in PLWHA, our data suggests it may be important to weigh the potential benefit of switching PLWHA to a HAART regimen with a lower associated risk of microcytosis in clinical decision-making among individuals in long-term HIV care. 

## Figures and Tables

**Table 1:** The description of Study Base by Type of Anemia present at enrollment in HIV-infected Ugandan Adults.

	No Anemia *n* = 170	Microcytic Anemia, *n* = 38	Macrocytic Anemia, *n* = 45	Anemia of Chronic Disease, *n* = 147	*p*-Value (Chi-square)
**Socio-demographic Factors**					
**Age**					0.64
18–29	44 (25.88%)	10 (26.32%)	14 (31.11%)	30 (20.41%)	
30–35	45 (26.47%)	7 (18.42%)	14 (31.11%)	38 (25.85%)	
36–41	39 (22.94%)	13 (34.21%)	9 (20.00%)	42 (28.57%)	
42+	42 (24.71%)	8 (21.05%)	8 (17.78%)	37 (25.17%)	
**Sex**					0.0044
Male	39 (22.94%)	11 (28.95%)	12 (26.67%)	61 (41.50%)	
Female	131 (77.06%)	27 (71.05%)	33 (73.33%)	86 (58.50%)	
**Wealth Quintile**					0.068
1	32 (18.82%)	7 (18.42%)	8 (17.78%)	33 (22.45%)	
2	32 (18.82%)	5 (13.16%)	6 (13.33%)	37 (25.17%)	
3	27 (15.88%)	15 (39.47%)	10 (22.22%)	28 (19.05%)	
4	41 (24.12%)	4 (10.53%)	8 (17.78%)	27 (18.37%)	
5	38 (22.35%)	7 (18.42%)	13 (28.89%)	22 (14.97%)	
**Work Status**					
No Income	22 (12.94%)	9 (23.68%)	5 (11.11%)	16 (10.88%)	0.0223
Informal economic activity	58 (34.12%)	12 (31.58%)	7 (15.56%)	51 (34.69%)	
Unskilled employment	50 (29.41%)	8 (21.05%)	17 (37.78%)	30 (20.41%)	
Driver/skilled laborer	13 (7.65%)	6 (15.79%)	7 (15.56%)	27 (18.37%)	
Professional	27 (15.88%)	3 (7.89%)	9 (20.00%)	23 (15.65%)	
**Education**					
Less than Primary	75 (44.12%)	17 (44.74%)	19 (44.22%)	55 (37.67%)	0.6189
Primary education complete	20 (11.76%)	5 (13.16%)	9 (20.00%)	20 (13.70%)	
Some Ordinary level	35 (20.59%)	8 (21.05%)	4 (8.89%)	32 (21.92%)	
Ordinary level or higher	40 (23.53%)	8 (21.05%)	13 (28.89%)	39 (26.71%)	
**Clinical Measures**					
High C-reactive protein	20 (11.98%)	10 (26.32%)	7 (15.56%)	18 (12.33%)	0.1722
**HAART regimen**					0.0876
D4T	1 (0.6)	0 (0)	0 (0)	2 (1.4)	
Efavirenz	30 (17.7)	7 (18.4)	9 (20.0)	25 (17.0)	
Nevirapine	57 (33.5)	18 (47.4)	14 (31.1)	37 (25.2)	
Naïve	82 (48.24%)	13 (34.21%)	22 (48.89%)	83 (56.46%)	
**Other Hematologic Indicators**					
WHO Anemia	0 (0.00%)	30 (78.95%)	17 (37.78%)	147 (100.00%)	<0.0001
Low Ferritin	36 (21.43%)	8 (21.05%)	9 (20.00%)	15 (10.20%)	0.0821
Normal Ferritin	75 (44.64%)	15 (39.47%)	17 (37.78%)	62 (42.18%)	
High Ferritin	57 (33.93%)	15 (39.47%)	19 (42.22%)	70 (47.62%)	
**Behavioral Factors**					
**Ever Smoked**					0.8339
Yes	32 (18.82%)	6 (15.79%)	6 (13.33%)	26 (17.69%)	
No	138 (81.18%)	32 (84.21%)	39 (86.67%)	121 (82.31%)	
**Current Alcohol use**					
Never Used	38 (22.35%)	10 (26.32%)	9 (20.00%)	27 (18.37%)	0.8941
Former User	94 (55.29%)	22 (57.89%)	27 (60.00%)	89 (60.54%)	
Current User	38 (22.35%)	6 (15.79%)	9 (20.00%)	31 (21.09%)	
**Baseline Reported Multivitamin Use**					0.1749
Yes	31 (18.24%)	10 (26.32%)	15 (33.33%)	34 (23.13%)	
No	139 (81.76%)	28 (73.68%)	30 (66.67%)	113 (76.87%)	
**Randomized to Multivitamins**					0.0292
No	95 (55.88%)	19 (50.00%)	14 (31.11%)	72 (48.98%)	
Yes	75 (44.12%)	19 (50.00%)	31 (68.89%)	75 (51.02%)	
**Vitamin D Deficiency**					0.0288
Deficient	24 (14.20%)	2 (5.26%)	11 (24.44%)	30 (20.55%)	
Insufficient	104 (61.54%)	26 (68.42%)	30 (66.67%)	81 (55.48%)	
Sufficient	41 (24.26%)	10 (26.32%)	4 (8.89%)	35 (23.97%)	
**Body Mass Index (BMI, kg/m^2^)**					0.0217
Underweight (BMI <18.5)	15 (8.82%)	0 (0.00%)	3 (6.67%)	4 (2.72%)	
Normal (18.5 < BMI < 25)	96 (56.47%)	29 (76.32%)	34 (75.56%)	104 (70.75%)	
Overweight (25 <BMI<30)	36 (21.18%)	5 (13.16%)	6 (13.33%)	25 (17.01%)	
Obese (30<BMI)	23 (13.53%)	4 (10.53%)	2 (4.44%)	14 (9.52%)	
**Outcome Measures**	Mean (SD)	Mean (SD)	Mean (SD)	Mean (SD)	*p*-value (ANOVA)
CD4 cell count	146.01 (105.41)	144.61 (83.40)	184.8 (93.67)	146.94 (97.11)	0.1107
Body Mass Index (kg/m^2^)	24.32 (4.45)	22.96 (2.91)	24.26 (5.20)	23.15 (4.26)	0.0588
Quality of Life Score	92.25 (8.88)	89.34 (9.70)	94.2 (10.85)	89.65 (10.60)	0.0121
Frailty Score	6.78 (2.88)	6.97 (2.86)	6.47 (2.90)	7.71 (3.25)	0.0203

**Table 2:** The temporal trends in the change of the Anemia type over the 12-month Follow-up: Entire Sample and by Baseline HAART Status.

	Month 0	Month 6	Month 12	Month 18
	Mean (SD)	Mean (SD)	Mean (SD)	Mean (SD)
**Entire Sample**	*n* = 400	*n* = 387	*n* = 372	*n* = 366
No anemia of any type	170 (42.5)	105 (27.1)	106 (28.5)	115 (31.4)
Microcytic Anemia	38 (9.5)	12 (3.1)	11 (3.0)	9 (2.5)
macrocytic anemia	45 (11.3)	198 (51.2)	200 (53.8)	195 (53.3)
ACD	147 (36.8)	72(18.6)	55 (14.8)	47 (12.8)
**HAART naïve at enrolment**	*n* = 200	*n* = 196	*n* = 178	*n* = 177
No anemia of any type	82 (41.0)	50 (25.5)	48 (27.0)	50 (28.3)
Microcytic Anemia	13 (6.5)	7 (3.6)	6 (3.4)	3 (1.7)
macrocytic anemia	22 (11.0)	108 (55.1)	102 (57.3)	102 (57.6)
ACD	83 (41.5)	31 (15.8)	22 (12.4)	22 (12.4)
**HAART Experienced at enrollment**	*n* = 200	*n* = 191	*n* = 194	*n* = 189
No anemia of any type	88 (44.0)	55 (28.8)	58 (29.9)	65 (34.4)
Microcytic Anemia	25 (12.5)	5 (2.6)	5 (2.6)	6 (3.2)
macrocytic anemia	23 (11.5)	90 (47.1)	98 (50.5)	93 (49.2)
ACD	64 (32.0)	41(21.5)	33 (17.0)	25 (13.2)

**Table 3:** The predictors of Incident Macrocytic anemia and Anemia of Chronic Disease over 18 months Follow-up among Ugandan adults free of anemia at enrollment.

	Incident Macrocytic Anemia	Incident Anemia of Inflammation (ACD)
	OR (95% CI)	OR (95% CI)
**Baseline HAART Regimen**		
Efavirenz Containing	0.423 (0.21, 0.85) *	1.39 (0.47, 4.10)
Nevirapine Containing	0.67 (0.38, 1.17)	0.10 (0.01, 0.87) *
HAART Naïve	Ref	Ref
Female vs. Male Sex	1.05 (0.53, 2.07)	3.61 (0.71, 18.3)
Age (per 5-year increment)	1.09 (0.94, 1.27)	1.04 (0.83, 1.29)
BCE vs. Placebo	1.86 (0.99, 3.51) **	0.47 (0.14, 1.68)
**Vitamin D Status**		
Deficient vs. Sufficient	1.09 (0.36, 3.34)	6.71 (0.52, 86.3)
Insufficient vs. Sufficient	0.99 (0.47, 2.11)	3.03 (0.32, 28.8)
Baseline CD4		
Per 100 cells/L	0.93 (0.46, 1.14)	0.47 (0.27, 0.79) *
≤100 vs. ≥201 cells/L	1.26 (0.70, 2.26)	2.24 (0.78, 6.46)
101–200 vs. ≥201 cells/L	1.04 (0.54, 2.01)	1.28 (0.30, 5.48)
**Baseline Ferritin**		
High vs. Normal	1.85 (1.03, 3.32) *	0.49 (0.16, 1.46)
Low vs. Normal	1.02 (0.47, 2.23)	0.71 (0.13, 3.91)
**Baseline BMI category**		
Underweight	0.54 (0.16, 1.77)	2.19 (0.52, 9.12)
Normal weight	Ref	Ref
Overweight	1.23 (0.65, 2.31)	0.24 (0.03, 1.84)
Obese	1.61 (0.76, 3.42)	1.39 (0.46, (4.15)
**Wealth** (per quintile increment)	*0.83 (0.67, 1.03)*	1.00 (0.67, 1.49)
Q1 (lowest) vs. Q5 (highest)	2.43 (0.94,6.28)	1.10 (0.17, 7.08)
Q2 vs. Q5 (highest)	2.47 (0.94, 6.46)	2.32 (0.49, 10.96)
Q3 vs. Q5 (highest)	1.66 (0.62, 4.41)	-
Q4 vs. Q5 (highest)	2.49 (1.01, 6.14) *	2.35 (0.50, 11.20)

Results are from multivariable analyses of anemia type (ACD, macrocytic, no-anemia) as a nominal multinomial outcome using SAS PROC GEE with no-anemia as reference logit category. The odds of incident ACD or macrocytic anemia in relationship to baseline sociodemographic (age, sex, wealth), nutritional status (randomization to BCE vs. placebo as part of parent trial, self-reported multivitamin use at enrolment, vitamin D status, BMI, ferritin), immune factors (CD4, CRP) and HAART status/regimen were estimated. *: Bolded Associations are statistically significant. **: Bolded and italicized associations are marginally significant.

**Table 4:** The baseline and Current Anemia type in relation to the change in absolute CD4 cell count, Body mass index, quality of life and frailty score Over 18 months *.

		Month 0	Month 6	Month 12	Month 18	*p*-Trend Group × Time
**Outcome**	**Exposure**	Mean Difference (95% CI)	Mean Difference (95% CI)	Mean Difference (95% CI)	Mean Difference (95% CI)	
CD4 cell-count	**Baseline Anemia Type**					<0.0001
	Microcytic Anemia	−6.79 (−38.4, 24.8)	−5.59 (−46.5, 35.31)	15.5 (−31.9, 62.8)	−14.3 (−53.7, 25.1)	
	Macrocytic Anemia	**41.2 (9.1, 73.2)**	−24.3 (−56.4, 7.8)	−3.84 (−40.0, 32.3)	−4.87 (−50.0, 40.3)	
	ACD **	3.8 (−18.6, 26.3)	10.4 (−19.0, 39.7)	−0.29 (−25.6, 25.0)	−7.4 (−36.0, 21.3)	
	No Anemia	Ref	Ref	Ref	Ref	
	**Current Anemia Type**					0.0077
	Microcytic Anemia	−4.62 (−32.6, 21.3)	−12.3 (−54.7, 30.2)	−4.1 (−61.8, 53.5)	57.8 (−3.5, 119.1)	
	Macrocytic Anemia	**52.7 (25.9, 79.6)**	12.6 (−6.15, 31.3)	−1.7 (−21.2, 17.8)	12.9 (−8.64, 34.37)	
	ACD **	1.22 (−17.7, 20.1)	10.9 (−19.3, 41.1)	−9.1 (−33.0, 14.9)	−20.3 (−49.4, 8.8)	
	No Anemia	Ref	Ref	Ref	Ref	
Body Mass Index	**Baseline Anemia Type**					0.0852
	Microcytic Anemia	**−0.50 (−0.98, −0.02)**	−0.20 (−0.85, 0.45)	0.54 (−0.19, 1.28)	0.66 (−0.67, 2.00)	
	Macrocytic Anemia	−0.33 (−1.03, 0.36)	0.05 (−0.21, 0.32)	0.05 (−0.26, 0.35)	−0.04 (−0.43, 0.35)	
	ACD	**−0.71 (−1.04, −0.38)**	**−0.33 (−0.64, −0.01)**	**−0.41 (−0.73, −0.09)**	−0.32 (−0.87, 0.22)	
	No Anemia	Ref	Ref	Ref	Ref	
	**Current Anemia Type**					0.0560
	Microcytic Anemia	**−0.51 (−0.98, −0.02)**	−0.27 (−0.93, 0.38)	0.68 (−0.06, 1.42)	0.81 (−0.50, 2.12)	
	Macrocytic Anemia	−0.33 (−1.03, 0.38)	0.05 (−0.21, 0.32)	0.03 (−0.28, 0.34)	−0.04 (−0.43, 0.35)	
	ACD	**−0.72 (−1.04, −0.38)**	**−0.35 (−0.67, −0.03)**	**−0.44 (−0.74, −0.13)**	−0.32 (−0.88, 0.23)	
	No Anemia	Ref	Ref	Ref	Ref	
Quality of Life	**Baseline Anemia Type**					0.1181
	Microcytic Anemia	**−3.66 (−6.76, −0.56)**	−3.68 (−8.75, 1.40)	−0.54 (−6.83, 5.75)	−1.49 (−11.36, 8.39)	
	Macrocytic Anemia	0.62 (−2.43, 3.67)	−1.76 (−3.63, 0.10)	−0.75 (−2.19, 0.69)	−1.00 (−2.59, 0.59)	
	ACD	**−2.97 (−4.89, −1.06)**	−1.19 (−3.27, 0.88)	**−3.24 (−5.94, −0.53)**	−2.31 (−5.44, 0.81)	
	No Anemia	Ref	Ref	Ref	Ref	
	**Current Anemia Type**					0.4860
	Microcytic Anemia	**−3.81 (−6.97, −0.67)**	−3.77 (−8.80, 1.25)	−0.92 (−7.18, 5.35)	−2.06 (−11.65, 7.53)	
	Macrocytic Anemia	0.45 (−2.60, 3.50)	−1.70 (−3.61, 0.21)	−0.82 (−2.31, 0.66)	−1.07 (−2.69, 0.56)	
	ACD	**−2.96 (−4.87, −1.05)**	−1.05 (−3.17, 1.07)	**−3.21 (−5.93, −0.49)**	−2.31 (−5.51, 0.88)	
	No Anemia	Ref	Ref	Ref	Ref	
Frailty	**Baseline Anemia Type**					0.3778
	Microcytic Anemia	0.48 (−0.37, 1.34)	1.15 (−0.70, 3.00)	−0.23 (−1.64, 1.17)	0.19 (−2.95, 3.33)	
	Macrocytic Anemia	−0.09 (−0.93, 0.74)	0.41 (−0.14, 0.95)	**0.56 (0.07, 1.05)**	0.06 (−0.42, 0.55)	
	ACD	**0.96 (0.34, 1.57)**	0.64 (−0.02, 1.30)	**1.16 (0.46, 1.86)**	0.56 (−0.30, 1.43)	
	No Anemia	Ref	Ref	Ref	Ref	
	**Current Anemia Type**					0.3616
	Microcytic Anemia	0.50 (−0.36, 1.36)	**1.26 (0.59, 3.12)**	−0.29 (−1.71, 1.13)	0.15 (−0.29, 3.29)	
	Macrocytic Anemia	−0.11 (−0.94, 0.71)	0.41 (−0.15, 0.96)	**0.55 (0.06, 1.04)**	0.08 (−0.40, 0.57)	
	ACD	**0.91 (0.29, 1.52)**	0.62 (−0.04, 1.28)	**1.16 (0.47, 1.85)**	0.57 (−0.30, 1.45)	
	No Anemia	Ref	Ref	Ref	Ref	

* For respective outcomes, multivariable model adjusted for: time, age, female sex, wealth, baseline CRP, ARV experience at enrollment, trial arm, multivitamin use history, baseline vitamin D, alcohol use and smoking status. Bolded Associations are statistically significant. ** ACD = Anemia of Chronic Disease.

**Table 5:** The time to post enrollment gain of at least 100 CD4 cells/L, hospitalization or death in relation to anemia type at the onset of antiretroviral therapy among adult persons living with HIV from Uganda.

		Events/Person-month	Unadjusted Association Hazard Ratio (95% CI)	Adjusted Association * Hazard Ratio (95% CI)
Time to post enrollment gain of > 100 cells/L	**Baseline Anemia Type**			
	Microcytic Anemia	31/465.2	1.20 (0.81, 1.78)	1.16 (0.77, 1.76)
	Macrocytic Anemia	24/688.0	0.55 (0.35, 0.854)	**0.57 (0.36, 0.89)**
	ACD	105/1693.5	1.05 (0.81, 1.37)	1.00 (0.76, 1.32)
	No Anemia	120/2059.9	Ref	Ref
	**Pattern of Change in Anemia Type**			
	ACD/microcytosis sustained in follow-up	60/1137.1	0.76 (0.51, 1.13)	**0.62 (0.40, 0.95)**
	Baseline ACD, resolved then macrocytosis	52/752.9	1.03 (0.69, 1.56)	0.92 (0.60, 1.41)
	Macrocytosis all intervals	22/658.3	0.45 (0.27, 0.76)	**0.43 (0.25, 0.73)**
	Incident ACD/microcytic anemia	9/253.5	0.50 (0.25, 1.04)	0.52 (0.25, 1.08)
	Incident macrocytosis	72/1221.4	0.85 (0.58, 1.25)	0.77 (0.52, 1.14)
	No anemia all intervals	42/658.6	Ref	Ref
Time to Hospitalization or Death	**Type of Anemia at baseline**			
	Microcytic Anemia	8/596.9	0.92 (0.43, 1.98)	0.73 (0.33, 1.62)
	Macrocytic Anemia	12/698.0	1.26 (0.66, 2.40)	1.25 (0.64, 2.46)
	Anemia of Chronic Disease	43/2296.0	1.30(0.84, 2.0)	1.10 (0.68, 1.78)
	No Anemia	39/2728.3	Ref	Ref
	**Change in Anemia Type**			
	ACD/microcytosis sustained in follow-up	7/364.8	1.47 (0.57, 3.79)	1.37 (0.52, 3.64)
	Baseline ACD, resolved then macrocytosis	29/1063.9	1.79 (0.90, 3.59)	1.40 (0.68, 2.92)
	Incident ACD/microcytic anemia	17/799.9	1.55 (0.73, 3.31)	1.40 (0.62, 3.18)
	Macrocytosis all intervals	17/1691.2	0.71 (0.33, 1.52)	0.62 (0.28, 1.37)
	Incident macrocytosis	25/1707.9	1.03 (0.50, 2.09)	0.98 (0.47, 2.05)
	No anemia all intervals	11/785	Ref	Ref

* Multivariable model adjusted for: age, female sex, wealth, baseline CRP, baseline weight, ARV experience at enrollment, trial arm, multivitamin use history, baseline vitamin D and smoking status. ACD = Anemia of Chronic Disease. Bolded Associations are statistically significant.

## References

[1] Mildvan D., Creagh T., Leitz G., Anemia Prevalence Study Group (2007). Prevalence of anemia and correlation with biomarkers and specific antiretroviral regimens in 9690 human-immunodeficiency-virus-infected patients: Findings of the Anemia Prevalence Study. Curr. Med. Res. Opin..

[2] Volberding P.A., Levine A.M., Dieterich D., Mildvan D., Mitsuyasu R., Saag M., Anemia in HIV Working Group (2004). Anemia in HIV infection: Clinical impact and evidence-based management strategies. Clin. Infect. Dis..

[3] Brentlinger P.E., Silva W.P., Vermund S.H., Valverde E., Buene M., Moon T.D. (2016). Practical management of HIV-associated anemia in resource-limited settings: Prospective observational evaluation of a new mozambican guideline. AIDS Res. Hum. Retroviruses.

[4] Woldeamanuel G.G., Wondimu D.H. (2018). Prevalence of anemia before and after initiation of antiretroviral therapy among HIV infected patients at Black Lion Specialized Hospital, Addis Ababa, Ethiopia: A cross sectional study. BMC Hematol..

[5] Kerkhoff A.D., Wood R., Cobelens F.G., Gupta-Wright A., Bekker L.G., Lawn S.D. (2014). Resolution of anaemia in a cohort of HIV-infected patients with a high prevalence and incidence of tuberculosis receiving antiretroviral therapy in South Africa. BMC Infect. Dis..

[6] Daka D., Lelissa D., Amsalu A. (2013). Prevalence of anaemia before and after the initiation of antiretroviral therapy at ART center of Hawassa University Referral Hospital, Hawassa, South Ethiopia. Sch. J. Med..

[7] Ezeamama A.E., Guwatudde D., Sikorskii A., Kabagambe E.K., Spelts R., Vahey G., Fenton J.I., Fawzi W.W. (2018). Impaired hematologic status in relation to clinical outcomes among HIV-infected adults from Uganda: A prospective cohort study. Nutrients.

[8] Enawgaw B., Alem M., Addis Z., Melku M. (2014). Determination of hematological and immunological parameters among HIV positive patients taking highly active antiretroviral treatment and treatment naive in the antiretroviral therapy clinic of Gondar University Hospital, Gondar, Northwest Ethiopia: A comparative cross-sectional study. BMC Hematol..

[9] Morris M.S., Jacques P.F., Rosenberg I.H., Selhub J. (2007). Folate and vitamin B-12 status in relation to anemia, macrocytosis, and cognitive impairment in older Americans in the age of folic acid fortification. Am. J. Clin. Nutr..

[10] Allain T.J., Gomo Z., Wilson A.O., Ndemera B., Adamchak D.J., Matenga J.A. (1997). Anaemia, macrocytosis, vitamin B12 and folate levels in elderly Zimbabweans. Cent. Afr. J. Med..

[11] Thoradeniya T., Wickremasinghe R., Ramanayake R., Atukorala S. (2006). Low folic acid status and its association with anaemia in urban adolescent girls and women of childbearing age in Sri Lanka. Br. J. Nutr..

[12] Kufel W.D., Hale C.M., Sidman E.F., Orellana C.E., Miller C.D. (2016). Nucleoside Reverse Transcriptase Inhibitor (NRTI) associated macrocytosis. Int. J. Virol. AIDS.

[13] Genne D., Sudre P., Anwar D., Goehring C., Saaidia A., Hirschel B., Study S.H.C. (2000). Causes of macrocytosis in HIV-infected patients not treated with zidovudine. J. Infect..

[14] Sternfeld T., Lorenz A., Schmid M., Schlamp A., Demmelmair H., Koletzko B., Bogner J.R. (2009). Increased red cell corpuscular volume and hepatic mitochondrial function in NRTI-treated HIV infected patients. Curr. HIV Res..

[15] Yu I., Greenberg R.N., Crawford T.N., Thornton A.C., Myint T. (2017). Persistence of Macrocytosis after Discontinuation of Zidovudine in HIV-Infected Patients. J. Int. Assoc. Provid. AIDS Care.

[16] Oliveira O.C., Oliveira R.A., Souza Ldo R. (2011). Impact of antiretroviral therapy on occurrences of macrocytosis in patients with HIV/AIDS in Maringa, State of Parana. Rev. Soc. Bras. Med. Trop..

[17] Berhane K., Karim R., Cohen M.H., Masri-Lavine L., Young M., Anastos K., Augenbraun M., Watts D.H., Levine A.M. (2004). Impact of highly active antiretroviral therapy on anemia and relationship between anemia and survival in a large cohort of HIV-infected women: Women’s Interagency HIV Study. J. Acquir. Immune. Defic. Syndr..

[18] Quiros-Roldan E., Castelli F., Lanza P., Pezzoli C., Vezzoli M., Biasiotto G., Zanella I., Inflammation in HIV Study Group (2017). The impact of antiretroviral therapy on iron homeostasis and inflammation markers in HIV-infected patients with mild anemia. J. Transl. Med..

[19] Bissinger R., Bouguerra G., Al Mamun Bhuyan A., Waibel S., Abbes S., Lang F. (2015). Efavirenz induced suicidal death of human erythrocytes. Cell Physiol. Biochem..

[20] Freercks R.J., Mehta U., Stead D.F., Meintjes G.A. (2006). Haemolytic anaemia associated with efavirenz. AIDS.

[21] Guwatudde D., Ezeamama A.E., Bagenda D., Kyeyune R., Wabwire-Mangen F., Wamani H., Mugusi F., Spiegelman D., Wang M., Manabe Y.C. (2012). Multivitamin supplementation in HIV infected adults initiating antiretroviral therapy in Uganda: The protocol for a randomized double blinded placebo controlled efficacy trial. BMC Infect. Dis..

[22] Stangl A.L., Bunnell R., Wamai N., Masaba H., Mermin J. (2012). Measuring quality of life in rural Uganda: Reliability and validity of summary scores from the medical outcomes study HIV health survey (MOS-HIV). Qual. Life Res..

[23] Ezeamama A.E., Woolfork M.N., Guwatudde D., Bagenda D., Manabe Y.C., Fawzi W.W., Smith Fawzi M.C. (2016). Depressive and anxiety symptoms predict sustained quality of life deficits in HIV-positive Ugandan adults despite antiretroviral therapy: A prospective cohort study. Medicine.

[24] Desquilbet L., Margolick J.B., Fried L.P., Phair J.P., Jamieson B.D., Holloway M., Jacobson L.P. (2009). Relationship between a frailty-related phenotype and progressive deterioration of the immune system in HIV-infected men. J. Acquir. Immune Defic. Syndr..

[25] Desquilbet L., Jacobson L.P., Fried L.P., Phair J.P., Jamieson B.D., Holloway M., Margolick J.B. (2007). HIV-1 infection is associated with an earlier occurrence of a phenotype related to frailty. J. Gerontol. A. Biol. Sci. Med. Sci..

[26] Kyeyune R., Saathoff E., Ezeamama A.E., Loscher T., Fawzi W., Guwatudde D. (2014). Prevalence and correlates of cytopenias in HIV-infected adults initiating highly active antiretroviral therapy in Uganda. BMC Infect. Dis..

[27] Ezeamama A.E., Guwatudde D., Wang M., Bagenda D., Brown K., Kyeyune R., Smith E., Wamani H., Manabe Y.C., Fawzi W.W. (2016). High perceived social standing is associated with better health in HIV-infected Ugandan adults on highly active antiretroviral therapy. J. Behav. Med..

[28] Jaganath D., Walker A.S., Ssali F., Musiime V., Kiweewa F., Kityo C., Salata R., Mugyenyi P., Trial D., Trial A. (2014). HIV-associated anemia after 96 weeks on therapy: Determinants across age ranges in Uganda and Zimbabwe. AIDS Res. Hum. Retroviruses.

[29] Ezeamama A.E., Guwatudde D., Wang M., Bagenda D., Kyeyune R., Sudfeld C., Manabe Y.C., Fawzi W.W. (2015). Vitamin-D deficiency impairs CD4+T-cell count recovery rate in HIV-positive adults on highly active antiretroviral therapy: A longitudinal study. Clin. Nutr..

[30] Guwatudde D., Wang M., Ezeamama A.E., Bagenda D., Kyeyune R., Wamani H., Manabe Y.C., Fawzi W.W. (2015). The effect of standard dose multivitamin supplementation on disease progression in HIV-infected adults initiating HAART: A randomized double blind placebo-controlled trial in Uganda. BMC Infect. Dis..

[31] Muma R.D., Ross M.W., Parcel G.S., Pollard R.B. (1995). Zidovudine Adherence among Individuals with Hiv-Infection. AIDS Care.

[32] Fischl M.A., Richman D.D., Grieco M.H., Gottlieb M.S., Volberding P.A., Laskin O.L., Leedom J.M., Groopman J.E., Mildvan D., Schooley R.T. (1987). The Efficacy of Azidothymidine (Azt) in the Treatment of Patients with Aids and Aids-Related Complex - a Double-Blind, Placebo-Controlled Trial. N. Engl. J. Med..

[33] Nagao T., Hirokawa M. (2017). Diagnosis and treatment of macrocytic anemias in adults. J. Gen. Fam. Med..

[34] Panwar A., Sharma S.C., Kumar N., Sharma A. (2016). A study of anemia in human immunodeficiency virus patients: Estimating the prevalence, analyzing the causative effect of nutritional deficiencies, and correlating the degree of severity with CD4 cell counts. Med. J. DY Patil. Vidyapeeth.

[35] Bain B.J. (1999). Pathogenesis and pathophysiology of anemia in HIV infection. Curr. Opin. Hematol..

[36] Kreuzer K.A., Rockstroh J.K. (1997). Pathogenesis and pathophysiology of anemia in HIV infection. Ann. Hematol..

[37] Woldeamanuel G.G., Wondimu D.H. (2018). Prevalence of thrombocytopenia before and after initiation of HAART among HIV infected patients at black lion specialized hospital, Addis Ababa, Ethiopia: A cross sectional study. BMC Hematol..

[38] Redig A.J., Berliner N. (2013). Pathogenesis and clinical implications of HIV-related anemia in 2013. Hematology Am. Soc. Hematol. Educ. Program.

